# Resveratrol improves the iron deficiency adaptation of *Malus baccata* seedlings by regulating iron absorption

**DOI:** 10.1186/s12870-021-03215-y

**Published:** 2021-09-23

**Authors:** Xiaodong Zheng, Huifang Chen, Qiufang Su, Caihong Wang, Guangli Sha, Changqing Ma, Zhijuan Sun, Xueqing Yang, Xiangyang Li, Yike Tian

**Affiliations:** 1 College of Horticulture, Qingdao Agricultural University, No. 700 Changcheng Road, Qingdao, 266109 China; 2 Qingdao Key Laboratory of Genetic Improvement and Breeding in Horticulture Plants, Qingdao, 266109 China; 3 Qingdao Academy of Agricultrual Science, Qingdao, 266109 China; 4 College of Life Science, Qingdao Agricultural University, Qingdao, 266109 China; 5 College of Plant Protection, Shenyang Agricultural University, Shenyang, 110866 China; 6 State Key Laboratory Breeding Base of Green Pesticide and Agricultural Bioengineering, Key Laboratory of Green Pesticide and Agricultural Bioengineering, Ministry of Education, Guizhou University, Guiyang, 550025 China

**Keywords:** *Malus baccata*, Resveratrol, Iron deficiency stress, Iron absorption

## Abstract

**Background:**

Resveratrol (Res), a phytoalexin, has been widely reported to participate in plant resistance to fungal infections. However, little information is available on its role in abiotic stress, especially in iron deficiency stress. *Malus baccata* is widely used as apple rootstock in China, but it is sensitive to iron deficiency.

**Results:**

In this study, we investigated the role of exogenous Res in *M. baccata* seedings under iron deficiency stress. Results showed that applying 100 μM exogenous Res could alleviate iron deficiency stress. The seedlings treated with Res had a lower etiolation rate and higher chlorophyll content and photosynthetic rate compared with the apple seedlings without Res treatment. Exogenous Res increased the iron content in the roots and leaves by inducing the expression of *MbAHA* genes and improving the H^+^-ATPase activity. As a result, the rhizosphere pH decreased, iron solubility increased, the expression of *MbFRO2* and *MbIRT1* was induced, and the ferric-chelated reductase activity was enhanced to absorb large amounts of Fe^2+^ into the root cells under iron deficiency conditions. Moreover, exogenous Res application increased the contents of IAA, ABA, and GA3 and decreased the contents of DHZR and BL for responding to iron deficiency stress indirectly. In addition, Res functioned as an antioxidant that strengthened the activities of antioxidant enzymes and thus eliminated reactive oxygen species production induced by iron deficiency stress.

**Conclusion:**

Resveratrol improves the iron deficiency adaptation of *M. baccata* seedlings mainly by regulating iron absorption.

**Supplementary Information:**

The online version contains supplementary material available at 10.1186/s12870-021-03215-y.

## Background

Iron (Fe) is one of the most essential micronutrients for plant growth, and it plays a vital role in several important physiological processes, such as chlorophyll biosynthesis, photosynthesis, and antioxidative defense [1, 2]. Although Fe is abundant in soil, it mainly exists in the form of low-bioavailability ferric iron (Fe^3+^), especially in alkaline calcareous soils where high bicarbonate contents reduce the availability of Fe severely [3]. About 30% of the world’s arable land is potentially Fe-deficient, and Fe deficiency stress is one of the widest-ranging abiotic stresses that constrain crop yield and quality [4].

Plants develop a series of mechanisms during their long-term struggle with environmental stresses. To cope with Fe deficiency stress, plants adopt two strategies for absorbing and translocating Fe [3, 5]. Plants that adopt Mechanism I usually include dicotyledons and non-gramineous monocots [6]. These plants absorb Fe mainly through three processes. First, the H^+^-ATPase (AHA) enzyme on the root plasma membrane secretes protons to decrease the rhizosphere pH for increasing Fe solubility. Second, ferric reductase oxidase 2 (FRO2) converts invalid Fe^3+^ to effective Fe^2+^. Third, the high-affinity Fe transporter (IRT1) transports Fe^2+^ into the root cells [6, 7]. Plants that adopt Mechanism II, including gramineous plants, synthesize and secrete mugineic acid family phytosiderophores (MAs) to form an MA-Fe^3+^ complex [3, 8]. Apple trees are dicotyledons, and they use Mechanism I to absorb and translocate Fe. At the transcriptional regulatory level, the Fer-like Fe deficiency-induced transcription factor (FIT), the core transcription factor, directly regulates the absorption of Fe [9]. Moreover, FIT can form a heterodimer with other basic Helix-Loop-Helix transcription factor (bHLH), including bHLH38/39/100/101, to control the ferric reduction response and Fe transport into the root by directly regulating the transcription of *FRO2* and *IRT1* genes under Fe deficiency stress [10]. In apples, MdbHLH104 is activated by the SUMO E3 ligase MdSIZ1 and targets *MdAHA8* to promote plasma membrane H^+^ exocytosis [5, 11].

Several studies have found that phytohormones are also involved in plants’ responses to Fe deficiency stress. Abscisic acid (ABA), gibberellin (GA), and salicylic acid (SA) help alleviate the damage caused by Fe deficiency stress, whereas jasmonate (JA), cytokinin (CTK), and brassinolide (BL) have the opposite effect [12–14]. Exogenous ABA application can considerably improve the shoot Fe content and recover the chlorosis phenotype [15]. SA can interact with NO to promote the absorption, transport, and activation of Fe in an Fe-deficient environment [16]. JA suppresses the Fe absorption of plant roots by inhibiting the expression of *IRT1* and *FRO2* [17]. Recent studies have shown that application of BL to rice can aggravate the symptoms of Fe deficiency [13]. The application of plant growth regulators is an effective approach for improving the Fe deficiency tolerance of crops because of the important role of phytohormones in plants’ response to Fe deficiency stress [15, 17].

Resveratrol (Res), a stilbenoid compound, has been identified in more than 70 plant species, including grapes, peanuts, and knotweed [18, 19]. Res is classified as an antimicrobial phytoalexin that contributes to plant response to biotic and abiotic stress [19]. Most of the studies on Res focused on improving its resistance to fungal infections. Romeropérez et al. [20]. demonstrated that the Res level in grape berries was significantly induced after infection by powdery mildew. Res could also help improve the resistance of apple leaves to *Venturia inaequalis* [21]. Overexpressing exogenous stilbene synthase (STS) genes, as the key enzyme in the Res synthesis pathway, in tobacco, wheat, rice, apple, and grape could enhance resistance to fungal pathogens [22–24]. With regard to the role of Res in plants’ response to abiotic stress, previous studies reported that Res content was induced by wounding or UV light [25]. Grimmig et al. [26] also found that Res production was related to plants’ response to ozone. In citrus seedlings, Res and its combination with α-tocopherol could mediate salt adaptation [27]. However, whether Res is involved in plants’ response to Fe deficiency and the underlying molecular and physiological mechanisms remain unknown.

Apple (*Malus domestica* Borkh.) is one of the most valuable horticultural fruit crops cultivated worldwide. However, one-third of apple orchards in China suffer from Fe deficiency stress with a significant chlorosis phenotype [28, 29]. Fe fertilizer is widely used in apple orchard production to supply deficient Fe^2+^, but long-term over-application of fertilizer can degrade soil biological characteristics in apple orchards and seriously affect the yield and quality of the fruit [30, 31]. Therefore, improving the utilization efficiency of Fe effectively and enhancing apple rootstock tolerance to Fe deficiency stress have become research hotspots. In this study, we investigated the effects of different concentrations of exogenous Res on *Malus baccata* seedlings under Fe deficiency stress. Then, we explored the potential physiological and molecular mechanisms through which Res influenced the photosynthetic system, Fe absorption, rhizosphere pH, antioxidase activity, and hormone content. The expression of the key function genes and the transcription factors related to Fe absorption in apple were also determined under Fe deficiency stress and exogenous Res treatment. Our findings can provide a theoretical basis for analyzing the mechanism of Res in apple under Fe deficiency stress.

## Materials and methods

### Plant materials and growth conditions

Seeds of apple (*Malus baccata*) purchased from Changjing Garden Seedling Farm of Shuyang County were sown in wet vermiculite after cold stratification. When the seedlings developed four leaves, they were transferred to pots with dimensions of 7 cm × 7 cm × 10 cm (length, width, and height) and irrigated with complete Hoagland’s nutrient solution. The apple seedlings were grown under the condition of 23 °C ± 2 °C in a 16/8 h light/dark cycle, and the light intensity was 100 μmol·m^−2^·s^−1^. After two weeks, the apple seedlings were used for Fe deficiency and exogenous Res treatment.

### Fe deficiency and exogenous Res treatment

A total of 180 apple seedlings with similar growth conditions were randomly divided into five groups. The seedlings in Group I, as the control, were watered with a complete nutrient solution (40 μM Fe). For the Fe deficiency treatment, the seedlings in Group II, III, IV, and V were all watered with Fe deficiency nutrient solution (4 μM Fe). In addition, the seedlings in Groups III, IV, and V were sprayed with 10, 100, and 200 μM of exogenous Res (Sangon, Shanghai, China), respectively. Res was dissolved in ethanol at a concentration of 10 mM and stored at − 20 °C. Res was sprayed every two days. The seedlings from all the groups were photographed, collected after Fe deficiency/Res treatment for 24 days, immediately frozen in liquid nitrogen, and stored at − 80 °C in a DW-86L388J ultra-cold storage freezer (Haier, Qingdao, China). The experiment was repeated three times.

For the nutrient solution culture of the apple seedlings. A total of 60 apple seedlings with similar growth conditions were randomly divided into three groups. The seedlings in Group I were cultured with complete nutrient solution (The iron concentration was 40 μM, pH = 5.9). For the Fe deficiency treatment, the seedlings in Group II and III were cultured with Fe deficiency nutrient solution (The iron concentration was 4 μM, pH = 5.9). In addition, Fe deficiency nutrient solution in Groups III were added with 100 μM of exogenous Res. After 10 days treatment, the etiolation rate and fresh weight were detected.

### Measurements of chlorophyll content and photosynthetic rate

Thirty apple seedlings were randomly selected from each group to measure the chlorophyll content and photosynthetic rate after Fe deficiency and exogenous Res treatment for 24 days. A SPAD-502 chlorophyll meter (Konica Minolta, Tokyo, Japan) was used for the determination of chlorophyll content. The photosynthetic rate was measured with an LI-6400XT meter (LI-COR, Lincoln, USA). The light intensity was set to 500 μmol·m^−2^·s^−1^ with 50% humidity. The temperature was 22 °C. Each experiment was independently repeated three times.

### Determination of Fe content

The apple seedlings from Groups I, II, and IV were collected for Fe content determination after 24 days of Fe deficiency and exogenous Res treatment. For the staining of Fe in roots, Perls staining was conducted using a Prussian blue Fe stain kit (Solarbio, Beijing, China). Samples were dehydrated at 105 °C for 30 min and baked at 80 °C for 72 h for the quantification of Fe content in roots and leaves. Then, the kiln-dried samples were digested with 12 mL of HNO_3_ and HClO_4_ and diluted with deionized H_2_O to 25 mL. Elemental analysis of Fe was performed via inductively coupled plasma–optical emission spectrometry (PerkinElmer, Waltham Massachusetts, USA) as described by Su et al. [32]. Each experiment was independently repeated three times.

### Rhizosphere pH staining

The roots from Groups I, II, and IV were collected for rhizosphere pH staining after 24 days of Fe deficiency and exogenous Res treatment. Rhizosphere pH staining was conducted as described by Zhao et al. [11]. Each experiment was independently repeated three times.

### Determination of root H^+^-ATPase and ferric-chelated reductase (FCR) activity

A total of 0.2 g of the roots of the *M. baccata* seedlings were collected after 24 days of Fe deficiency and exogenous Res treatment for the detection of root H^+^-ATPase and FCR activity by using H^+^-ATPase and FCR activity extraction kits, respectively (Suzhou Geruisi Biotechnology Co., Ltd., Suzhou, China). Each experiment was independently repeated three times.

### Measurements of reactive oxygen species (ROS) level and malondialdehyde (MDA) content

The leaves in Groups I, II, and IV after Fe deficiency and exogenous Res treatment for 24 days were used to determine the ROS level and MDA content. Dying of superoxide anions (O_2_·^−^) and hydrogen peroxide (H_2_O_2_) contents was conducted by following the procedure of Zheng et al. [33]. MDA content was detected using a plant MDA extraction kit (Nanjing Jiancheng Bioengineering Institute, Nanjing, China). Each experiment was independently repeated three times.

### Detection of antioxidant enzyme activities

A total of 0.2 g of apple leaves were ground in phosphate buffer with a concentration of 100 mM (pH 7.4), transferred into 1.5 mL tubes afterward, and centrifuged at 4,000 × *g* for 10 min at 4 °C. The supernatants were used for the detection of SOD, POD, and CAT enzyme activities at the absorbance of 560, 470, and 240 nm, respectively. Enzyme activities were detected using plant SOD, POD, and CAT extraction kits (Nanjing Jiancheng Bioengineering Institute, Nanjing, China). Each experiment was independently repeated three times.

### Determination of electrolyte leakage and osmolytes

A total of 0.3 g of the leaves of the *M. baccata* seedlings were collected after 24 days of Fe deficiency and exogenous Res treatment for the detection of electrolyte leakage and osmolytes, respectively. Electrolyte leakage was determined as described by Su et al. [33]. Osmolytes were determined with plant proline, soluble sugar, and soluble protein extraction kits (Suzhou Geruisi Biotechnology Co., Ltd., Suzhou, China). Each experiment was independently repeated three times.

### Measurement of endogenous hormone content

A total of 0.5 g of the leaves of the *M. baccata* seedlings were collected after 24 days of Fe deficiency and exogenous Res treatment for the detection of endogenous hormones, including IAA, GA3, ABA, DHZR, BL, and JA-Me. Measurement of the endogenous hormone content was performed using electrospray ionization–high-performance liquid chromatography–tandem mass spectrometry in accordance with the method described by Zhuo et al. [34]. Each experiment was independently repeated three times.

### Quantitative RT-PCR (RT-qPCR) assay

Total RNA was extracted from the roots of the *M. baccata* seedlings by using a FastPure Plant Total RNA Isolation Kit (Vazyme, Nanjing, China), and the cDNA was synthesized from 2 μg of total RNA by using 5 × All-In-One RT MasterMix (ABM, Sydney, Australia). LightCycler® 480 SYBR Green Master (Roche, Mannheim, Germany) with a LightCycler® 480 II system (Roche, Rotkreuz, Switzerland) was used for the qPCR assay, and the primers are listed in Table S1. The primer sequences for qPCR were designed in accordance with the coding sequence of genes in Primer 5 software and checked using a BLAST search in the apple genomic database. The relative expression was calculated with the 2^−∆∆Ct^ method. Each experiment was independently repeated three times.

### Statistical analysis

The data were subjected to ANOVA followed by Fisher’s LSD or Student’s *t-*test analysis. Statistically significant differences were indicated by *P* < 0.05. Statistical computations were conducted using SPSS software (IBM, Armonk, NY, USA).

## Results

### Effects of exogenous Res on apple seedlings under Fe deficiency stress

**Fig. 1:**
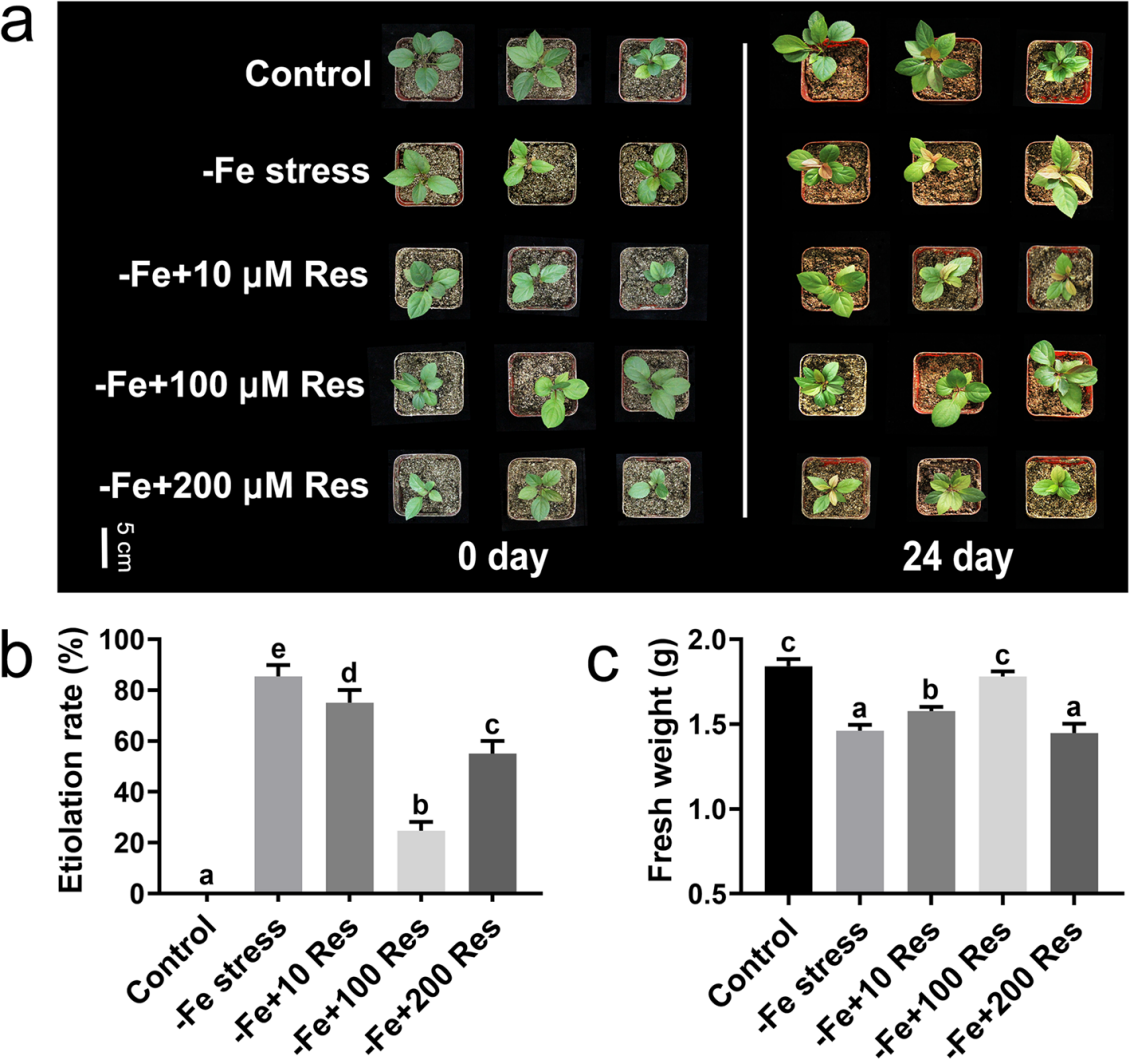
Effects of exogenous Res on apple seedlings under Fe deficiency stress. **a** The phenotype resulting from the application of different concentrations of exogenous Res (10, 100, 200 μM) to *Malus baccata* seedlings under Fe deficiency stress at day 0 and day 24. The etiolation rate (**b**) and fresh weight (**c**) of the apple seedlings after Fe deficiency and exogenous Res treatment for 24 days. Data represent the means ± SD of triplicate experiments. Different lowercase letters indicate significant differences, according to Fisher’s LSD (*P* < 0.05)

Res with different concentrations was sprayed on the leaves of the apple seedlings under Fe deficiency stress to determine the effects of exogenous Res on the resistance of *M. baccata* under Fe deficiency stress. As shown in Fig. 1A, the young leaves of the apple seedlings exhibited serious leaf chlorosis under Fe deficiency stress for 24 days compared with the control. However, different concentrations of exogenous Res could alleviate the chlorosis to different degrees. When low-concentration (10 μM) and high-concentration (200 μM) Res were applied to the apple seedlings under Fe deficiency, the young leaves were greener than the leaves of the stressed seedlings and had a much lower etiolation rate than the control (Fig. 1B). When 100 μM Res was applied, the young leaves maintained normal growth and had the lowest etiolation rate (24.67%) compared with the seedlings with Fe deficiency stress (85.33%) (Fig. 1B). Moreover, the fresh weight of the young leaves under Fe deficiency stress decreased considerably from 1.84 g to 1.46 g. When 100 μM Res was applied, the fresh weight recovered to 1.78 g (Fig. 1C). In addition, we also explored the effect of 100 μM exogenous Res on apple seedlings under Fe deficiency stress in nutrient solution. As shown in Figure S1A, the young leaves of the apple seedlings under Fe deficiency stress for 10 days were yellowing, while the seedlings after exogenous Res treatment were still kept green. In addition, the etiolation rate of the apple seedlings after Res treatment for 10 days (21%) was significantly lower than that without Res treatment (70%) under Fe deficiency stress (Fig. S1B). Moreover, the fresh weight of the seedlings after Res treatment was also significantly higher than that without exogenous Res treatment (Fig. S1C). Therefore, 100 μM of Res was selected for further research.

### Effects of exogenous Res on chlorophyll content and photosynthetic rate under Fe deficiency stress

**Fig. 2:**
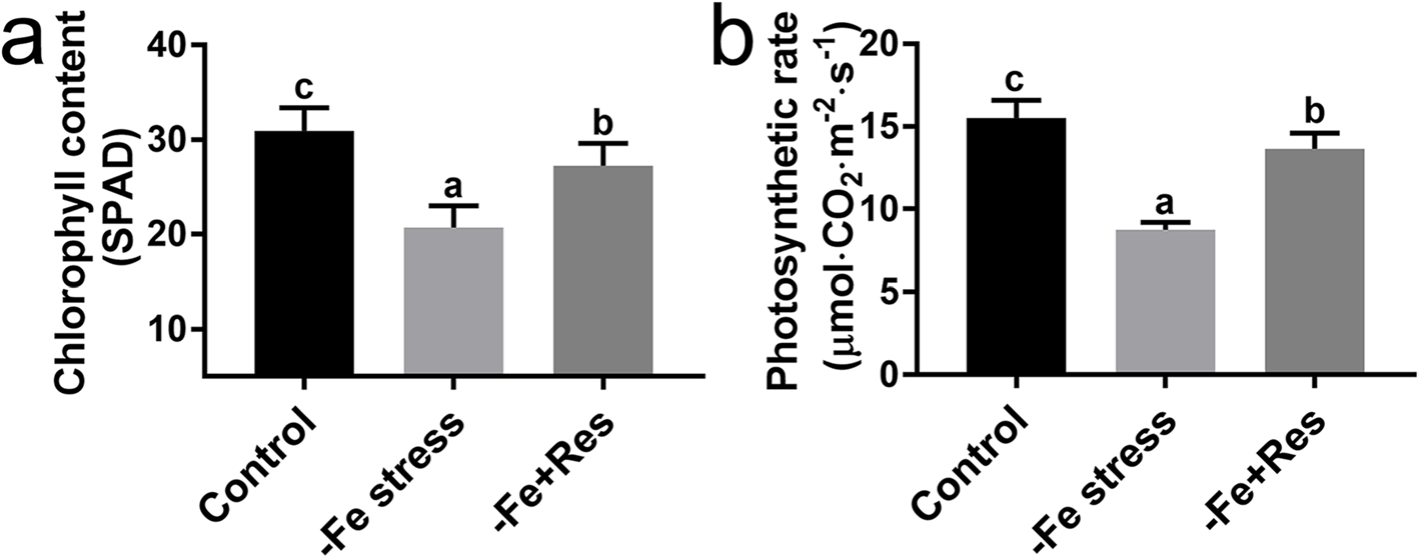
Effects of exogenous Res on chlorophyll content and photosynthetic rate under Fe deficiency stress. The chlorophyll content (**a**) and photosynthetic rate (**b**) of the apple seedlings after Fe deficiency and exogenous Res treatment for 24 days. Data represent the means ± SD of triplicate experiments. Different lowercase letters indicate significant differences, according to Fisher’s LSD (*P* < 0.05)

The chlorophyll content and photosynthetic rate of the apple seedlings were also determined to examine the chlorosis phenotype caused by Fe deficiency stress. The chlorophyll content decreased from 29.9 SPAD to 20.7 SPAD after 24 days of Fe deficiency stress. However, the application of exogenous Res alleviated the decline in chlorophyll content, which recovered to as high as 27.2 SPAD (Fig. 2A). Similarly, the photosynthetic rate of the apple seedlings dramatically decreased from 15.5 μmol·m^−2^·s^−1^ to 8.7 μmol·m^−2^·s^−1^ after Fe deficiency stress. When exogenous Res was applied, the photosynthetic rate of the apple seedlings improved by 55.9% and reached 13.6 μmol·m^−2^·s^−1^ (Fig. 2B). These results showed that the application of exogenous Res effectively increased the chlorophyll content and photosynthetic rate under Fe deficiency stress.

### Effects of exogenous Res on Fe content under Fe deficiency stress

**Fig. 3:**
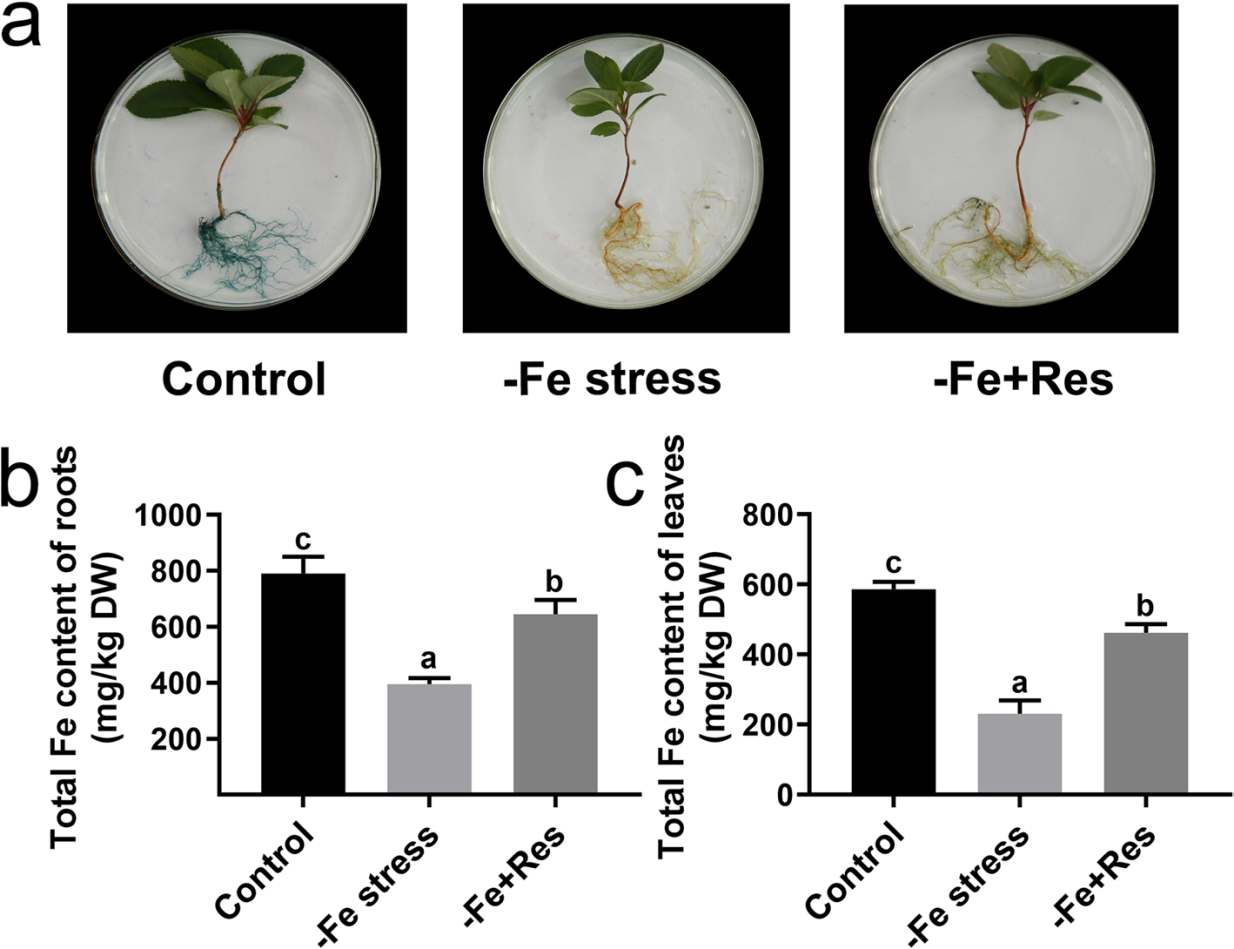
Effects of exogenous Res on Fe content under Fe deficiency stress. **a** Perls staining of Fe in roots. Total Fe content of roots (**b**) and leaves (**c**) after Fe deficiency and exogenous Res treatment for 24 days. DW means dry weight. Data represent the means ± SD of triplicate experiments. Different lowercase letters indicate significant differences, according to Fisher’s LSD (*P* < 0.05)

Perls staining was performed to detect the Fe content in roots and explore whether exogenous Res could affect the Fe content under Fe deficiency stress. The Fe content under Fe deficiency stress was much lower than that in the control, and the staining results were considerably shallower. However, exogenous Res significantly enhanced the Fe content under Fe deficiency stress and produced deep staining results (Fig. 3A). These staining results are consistent with the results of the quantification of Fe contents in the roots. The Fe content in the roots decreased from 790.22 mg/kg DW to 491.88 mg/kg DW after Fe deficiency stress for 24 days. When exogenous Res was applied, the Fe content increased to 644.51 mg/kg DW (Fig. 3B). Aside from the Fe content in the roots, the Fe content in the leaves was also investigated. The Fe content in the leaves decreased from 586.51 mg/kg DW to 231.22 mg/kg DW under Fe deficiency stress, but exogenous Res increased this content to as high as 462.47 mg/kg DW (Fig. 3C). These results indicated that the application of exogenous Res increased the Fe content in the roots and leaves under Fe deficiency stress.

### Effects of exogenous Res on rhizosphere pH and FCR activity under Fe deficiency stress

**Fig. 4:**
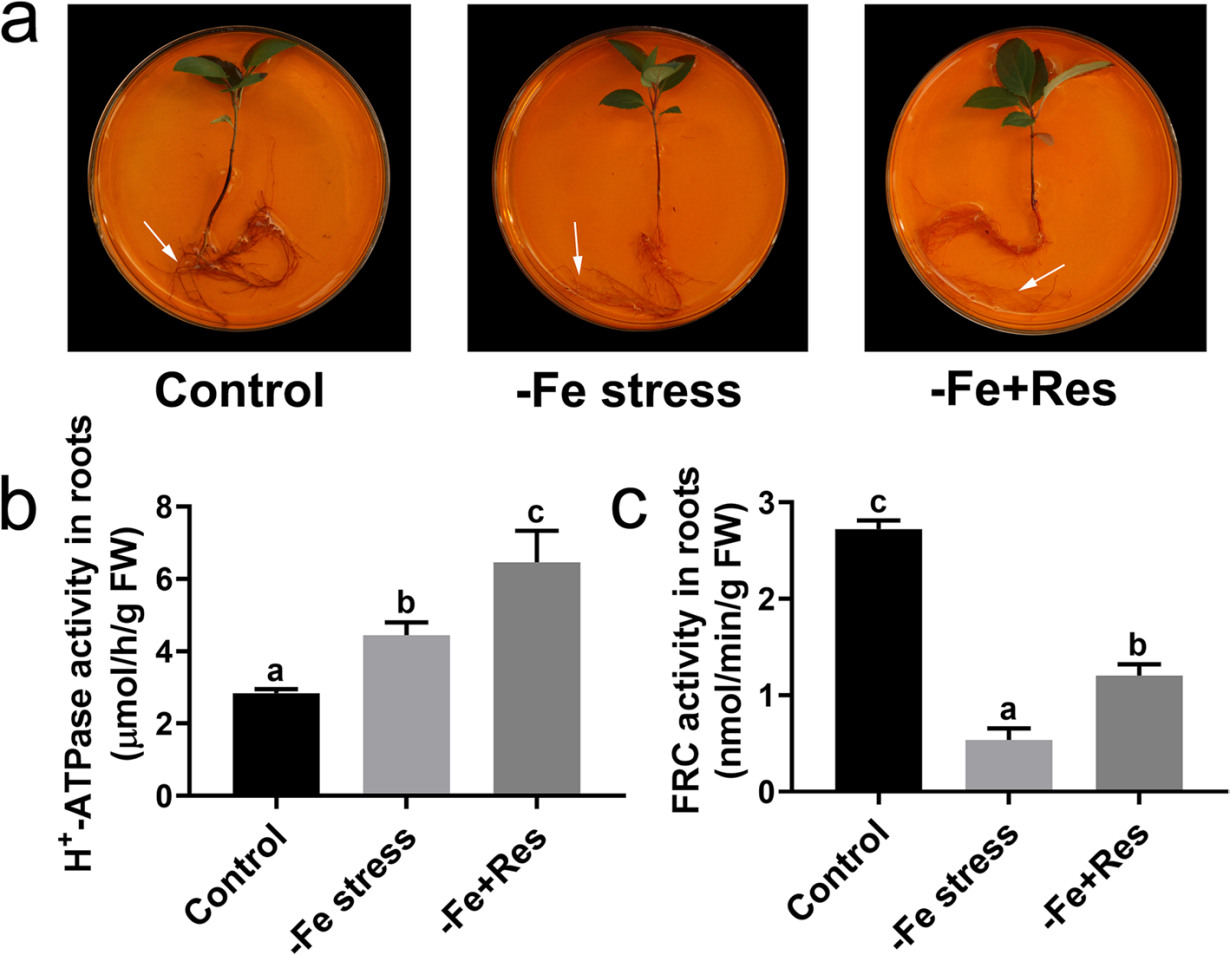
Effects of exogenous Res on rhizosphere pH and FCR activity under Fe deficiency stress. **a** The rhizosphere pH staining of the roots after Fe deficiency and exogenous Res treatment for 24 days. The arrows indicated the rhizosphere. The activities of H^+^-ATPase (**b**) and FCR (**c**) in roots after Fe deficiency and exogenous Res treatment for 24 days. FW means fresh weight. Data represent the means ± SD of triplicate experiments. Different lowercase letters indicate significant differences, according to Fisher’s LSD (*P* < 0.05)

Rhizosphere pH staining was performed to explore the mechanisms of the positive effect of exogenous Res on Fe deficiency stress. The results showed that the rhizosphere pH stain was very shallow under Fe deficiency stress. When exogenous Res was applied, the rhizosphere pH stain became even shallower than that under Fe deficiency stress (Fig. 4A). Furthermore, the activity of root H^+^-ATPase was determined. The results showed that the H^+^-ATPase activity increased under Fe deficiency stress from 2.84 μmol/h/g FW to 4.44 μmol/h/g FW. Exogenous Res could even enhance the H^+^-ATPase activity to 6.46 μmol/h/g FW (Fig. 4B). In addition, FCR activity was examined. Unlike H^+^-ATPase activity, FCR activity decreased significantly under Fe deficiency stress from 2.72 nmol/min/g FW to 0.54 nmol/min/g FW. However, when exogenous Res was applied, FCR activity was induced and reached 1.20 nmol/min/g FW (Fig. 4C).

### Effects of exogenous Res on oxidative damage and antioxidant enzyme activities under Fe deficiency stress

The oxidative damage induced by Fe deficiency stress was determined. As shown in Fig. S2A, the O_2_·^−^ and H_2_O_2_ contents were considerably increased by Fe deficiency stress. However, exogenous Res could decrease the O_2_·^−^ and H_2_O_2_ levels. The content of MDA, as the indicator of lipid peroxidation damage, under Fe deficiency stress was 44.17% higher than that of the control group. When exogenous Res was applied, the MDA content recovered to the normal level (Fig. S2B).

The activities of antioxidant enzymes SOD, POD, and CAT were also examined to determine the role of Res in antioxidant enzymes under Fe deficiency stress. A similar variation tendency was exhibited by the three antioxidant enzymes. The activities of SOD, POD, and CAT were significantly inhibited under Fe deficiency stress. However, exogenous Res could recover the activities of the three antioxidant enzymes, especially CAT (Fig. S2C–E). The CAT activity after Fe deficiency stress for 24 days was only 6.29 U/mg protein, which is much lower than that of the control group (11.46 U/mg protein). Exogenous Res application could increase such activity by 68.20% to as high as 10.58 U/mg protein (Fig. S2E). These results demonstrated that the application of exogenous Res alleviated the oxidative damage under Fe deficiency stress.

### Effects of exogenous Res on electrolyte leakage and osmolytes under Fe deficiency stress

**Fig. 5:**
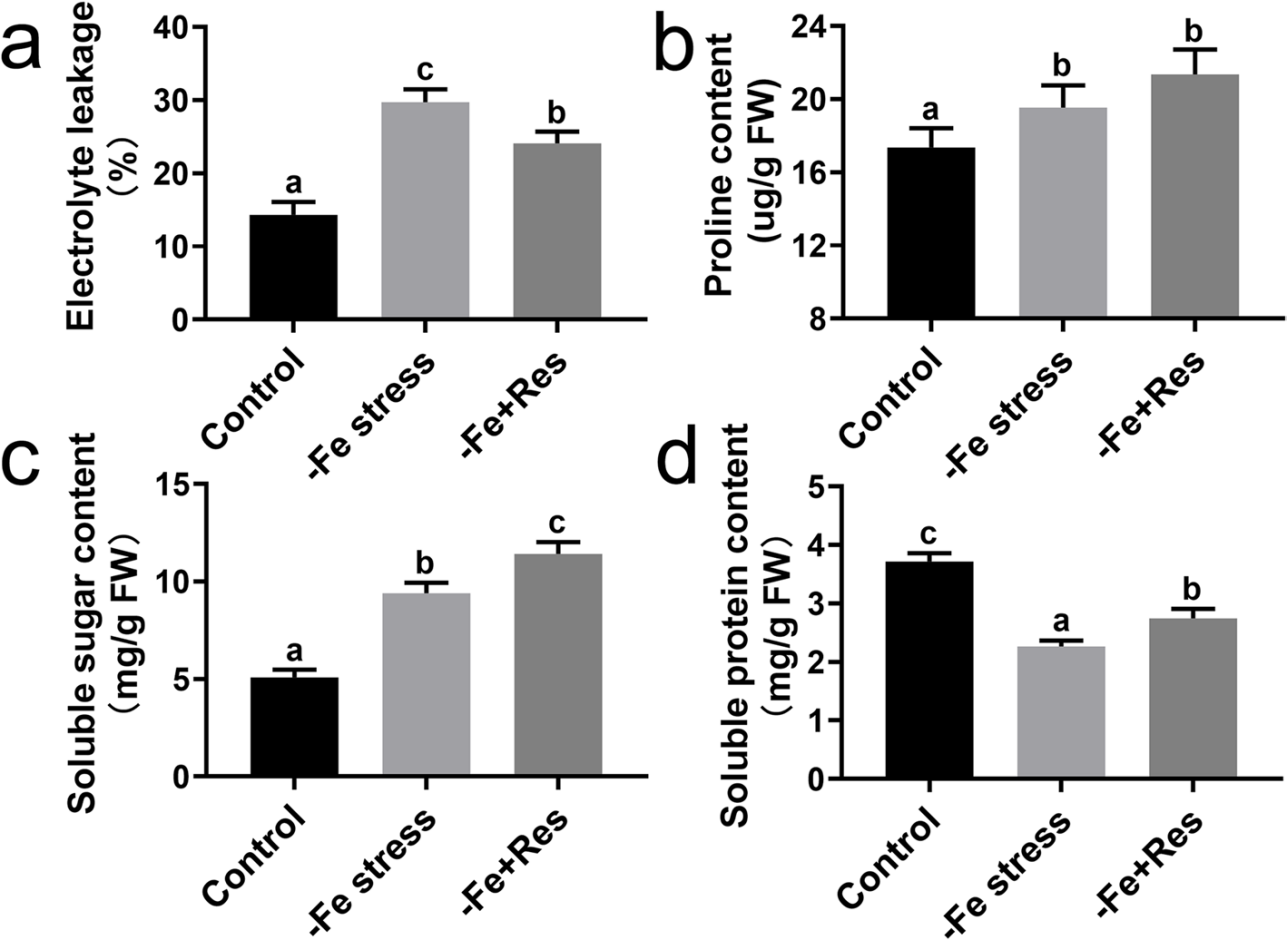
Effects of exogenous Res on electrolyte leakage and osmolytes under Fe deficiency stress. Effects of exogenous Res application on electrolyte leakage (**a**), proline content (**b**), soluble sugar content (**c**) and soluble protein content (**d**) under Fe deficiency stress. FW means fresh weight. Data represent the means ± SD of triplicate experiments. Different lowercase letters indicate significant differences, according to Fisher’s LSD (*P* < 0.05)

Electrolyte leakage was determined under Fe deficiency stress. Electrolyte leakage was significantly increased by Fe deficiency stress from 14.3% to 29.7%. When 100 μM exogenous Res was applied, the electrolyte leakage was reduced to 24.07% (Fig. 5A).

The contents of osmolytes, including proline, soluble sugar, and soluble protein, were also measured. Fe deficiency stress significantly increased the soluble sugar and proline contents but decreased the soluble protein content. When exogenous Res was applied, the contents of soluble sugar and soluble protein increased significantly, but the content of proline exhibited almost no change (Fig. 5B–D).

### Effects of exogenous Res on endogenous hormone content under Fe deficiency stress

**Fig. 6:**
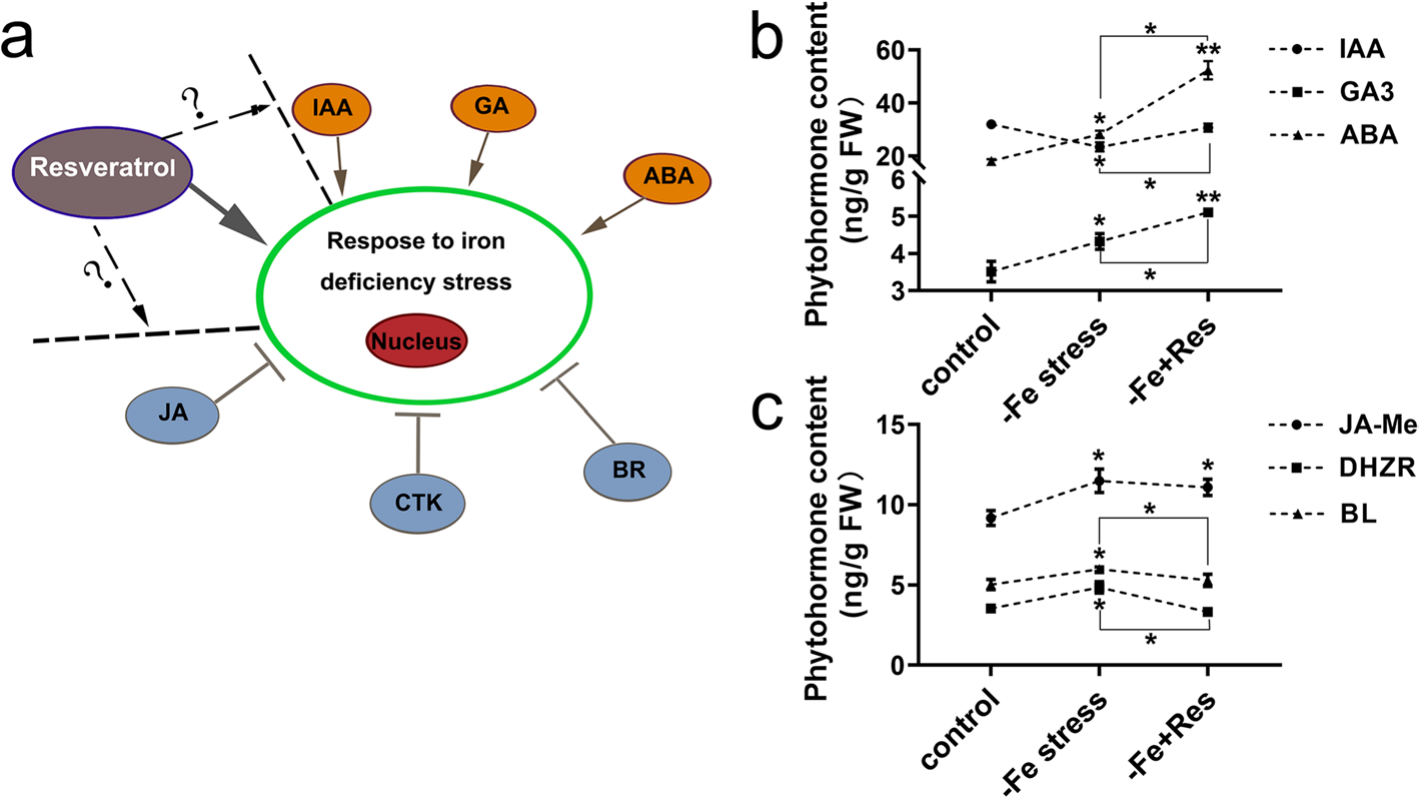
Effects of exogenous Res on endogenous hormone contents under Fe deficiency stress. **a** Mechanisms of hormones response to Fe deficiency stress. Effects of exogenous Res on IAA, GA3, ABA contents (**b**) and JA-Me, DHZR, BL contents (**c**) under Fe deficiency stress. FW means fresh weight. Data are means ± SD of triplicate experiments. Asterisks (*) indicate significant differences from the control (Student’s t-test, *P < 0.05, **P < 0.01)

Previous studies have shown that plant hormones are involved in plants’ response to Fe deficiency stress. IAA, GA, and ABA play a positive role, whereas CTK, JA, and BR play a negative role. However, the effect of exogenous Res on endogenous hormone content under Fe deficiency has never been reported (Fig. 6A). Thus, the endogenous hormone contents under Fe deficiency stress and exogenous Res treatment were examined in this study.

Under Fe deficiency stress, the contents of ABA and GA3 increased significantly, whereas the IAA content was inhibited. When exogenous Res was applied, the contents of IAA, ABA, and GA3 increased by 31.04%, 85.73%, and 18.10%, respectively (Fig. 6B). The contents of JA-Me, DHZR, and BL also increased under Fe deficiency stress. When exogenous Res was applied, the levels of DHZR and BL decreased from 4.85 ng/g to 3.32 ng/g and from 5.98 ng/g to 5.29 ng/g, respectively. The content of JA-Me exhibited no significant change (Fig. 6C).

### Effects of exogenous Res on the expression of Fe-deficiency responding genes

To elucidate the mechanism of exogenous Res involvement in the Fe deficiency response, we performed qPCR to detect the expression of Fe-related genes under Fe deficiency stress and exogenous Res treatment.

**Fig. 7:**
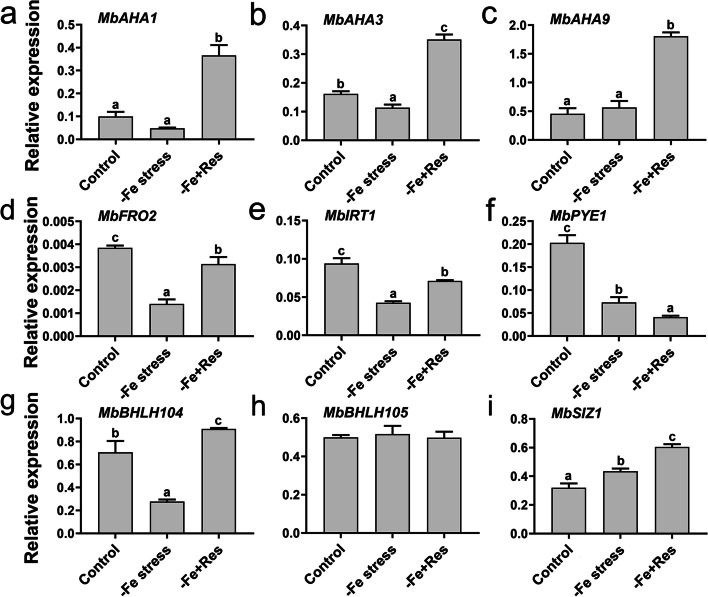
Effects of exogenous Res on the expression of Fe deficiency responding genes in roots. The expression of *MbAHA1* (**a**), *MbAHA3* (**b**), *MbAHA9* (**c**), *MbFRO2* (**d**), *MbIRT1* (**e**), *MbPYE1* (**f**), *MbBHLH104* (**g**), *MbBHLH105* (**h**), and *MbSIZ1* (**i**) in roots after Fe deficiency and exogenous Res treatment for 24 days. Data represent the means ± SD of triplicate experiments. Different lowercase letters indicate significant differences, according to Fisher’s LSD (*P* < 0.05)

As shown in Fig. 7, exogenous Res significantly upregulated the expression of *MbAHA1*, *MbAHA3*, and *MbAHA9*, as H^+^-ATPase (AHA) enzyme family genes, by 6.63, 2.09, and 2.19 times, respectively (Fig. 7A–C). The expression of *MbFRO2* and *MbIRT1* was significantly downregulated under Fe deficiency stress. However, when exogenous Res was applied, the expression of *MbFRO2* and *MbIRT1* increased by 1.24 and 0.67 times, respectively (Fig. 7D and E). For the bHLH-type transcription factors, the expression of *MbPYE* and *MbBHLH104* was significantly downregulated under Fe deficiency stress. When exogenous Res was applied, the expression of *MbPYE* was continuously suppressed, whereas the expression of *MbBHLH104* was induced (Fig. 7F and G). Meanwhile, the expression of *MbBHLH105* had no significant change under Fe deficiency stress and exogenous Res treatment (Fig. 7H). For the SUMO E3 ligase protein, the expression of *MdSIZ1* was significantly upregulated by Fe deficiency stress, and exogenous Res could further increase its expression (Fig. 7I).

## Discussion

Fe deficiency is one of the most widespread micronutrient deficiency stresses faced by plants, and it severely limits crop quality and yield [28, 35]. *M. baccata*, which is native to the Greater Higgnan Mountains, is widely used as an apple rootstock in northern China [36]. It has high resistance to low temperature and can be used as a raw material for breeding cold-tolerant apple rootstock. However, *M. baccata* is sensitive to Fe deficiency stress [37, 38]. Therefore, the Fe deficiency tolerance of *M. baccata* seedlings must be enhanced. The application of plant growth regulators is an effective approach for improving the Fe deficiency tolerance of crops [15, 17]. Res is a phytoalexin that contributes to plant biotic responses [19, 21]. Exogenous application of Res reduces postharvest decay in several fruit types, such as tomatoes, grapes, and avocado pears [39]. However, its role in plants’ response to Fe deficiency stress has never been reported. In this study, we investigated the role of different concentrations of Res in *M. baccata* seedlings under Fe deficiency stress. The effect of applying 100 μM of Res on Fe deficiency tolerance was much better than the effect of applying 10 μM (low concentration) and 200 μM (high concentration) of Res; it also produced the lowest etiolation rate and highest fresh weight (Fig. 1). Plant growth regulators usually affect plant growth and development in a dose-dependent manner. Su et al. [32] reported that the effect of applying 0.2 mg/L of BL on improving the salt tolerance of apple seedlings was much better than the effect of applying BL concentrations of 0.05 and 1.0 mg/L. In *M. baccata* seedlings, 600 μM for irrigation or 200 μM for spraying was selected as the best concentration of melatonin to maximize its role under waterlogging stress [33]. Our result indicated that 100 μM Res was an appropriate concentration for enhancing the Fe deficiency tolerance of *M. baccata* seedlings.

In higher plants, most of the leaf Fe is located in the chloroplasts, and abundant Fe is essential for maintaining the function of the chloroplasts and photosynthetic system [6, 40]. Chlorophyll is the major component of chloroplasts; Fe deficiency can lead to a reduction in chlorophyll content and photosynthetic rate, which causes the yellowing leaf phenotype [41]. In this study, Fe deficiency decreased the leaf chlorophyll content and photosynthetic rate of the apple seedlings. However, the application of exogenous Res increased the chlorophyll content and photosynthetic rate under Fe deficiency stress, thereby alleviating leaf chlorosis (Fig. 2). To investigate how exogenous Res protected the photosynthetic system under Fe deficiency stress, the Fe contents in the roots and leaves under Fe deficiency stress and exogenous Res treatment were detected. Our results indicated that exogenous Res significantly increased the Fe content in the leaves and roots under Fe deficiency stress. This result was consistent with the Fe staining result (Fig. 3). These results indicated that exogenous Res exerted a positive effect on the absorption of Fe and protected the chlorophyll and photosynthetic system from Fe deficiency stress. This was the novel role of Res in plants’ response to Fe deficiency stress.

We analyzed how Res enhances Fe uptake in Fe-deficient apple seedlings via the mechanism of plant response to Fe deficiency stress. Apples are dicotyledons that adopt the typical Mechanism I strategy to absorb and translocate Fe [5, 42]. Under Fe deficiency stress, first, plants develop a proton pump that acidifies the rhizosphere to increase the solubility of Fe through the upregulation of plasma membrane (PM) H^+^-ATPase (AHA) family genes, such as *CsAHA1*, *AtAHA2*, and *MdAHA8* [11, 38]. In the present study, we found that rhizosphere pH was significantly reduced, whereas the activity of H^+^-ATPases increased after exogenous Res was applied under Fe deficiency stress (Fig. 4). Moreover, exogenous Res application significantly enhanced the expression of the *MbAHA* family, including *MbAHA1*, *MbAHA3*, and *MbAHA9* (Fig. 7). These results indicated that Res increased the amount of soluble Fe by acidifying the rhizosphere and upregulating the expression of *MbAHA* family genes. Second, FRO2 and ferric-chelated reductase convert Fe^3+^ to Fe^2+^, which is then transported into the roots via IRT1 [5–7]. Our results indicated that exogenous Res significantly increased the activity of FCR and induced the expression of *MbFRO2* and *MbIRT1* (Figs. 4 and 7). Therefore, we speculated that exogenous Res could increase the expression of *MbFRO2* and *MbIRT1* and enhance the activity of FCR to utilize the soluble Fe in the rhizosphere and transport Fe^2+^ to cells under Fe deficiency stress. Third, transcriptional regulation is one of the most common ways to regulate the function of genes involved in Fe uptake [5, 9, 10]. bHLH TFs have been reported to regulate the Fe deficiency response [6]. AtbHLH104 and AtbHLH105 positively regulate Fe absorption and rhizosphere acidification by directly activating the transcription of *bHLH38/39/100/101* [43]. Fe transport-related genes and *FRO3* are up-regulated in *pye-1* mutant under Fe-deficient conditions, suggesting that the PYE bHLH protein functions as a negative regulator under Fe deficiency stress in *Arabidopsis* [44]. In apple, MdbHLH104 has been identified as a positive regulator by directly binding to the promoter of *MdAHA8* to activate PM H^+^-ATPase activity and regulate rhizosphere acidification under Fe deficiency stress [11]. In the present study, exogenous Res significantly increased the expression of *MbbHLH104* and decreased the expression of *MbPYE1* under Fe deficiency stress. Meanwhile, no significant expression change was observed for *MbbHLH105* (Fig. 7). In addition, MdSIZ1 enhances the stability of MdbHLH104 protein and promotes PM H^+^ exocytosis and rhizosphere acidification in the Fe deficiency response [5]. Our results also showed that exogenous Res significantly increased the expression of *MbSIZ1* under Fe deficiency stress (Fig. 7). These results were consistent with the result that a high Fe content was detected in the apple seedlings with exogenous Res treatment under Fe deficiency stress (Fig. 3). Therefore, we inferred that exogenous Res could enhance the Fe uptake under Fe deficiency stress mainly by regulating the signal response genes in the Mechanism I strategy.

Fe is an essential component of electron transport chains in mitochondria and chloroplasts. Fe deficiency stress can induce oxidative damage [45]. In the present study, we investigated the influence of Fe deficiency stress on ROS level and MDA content. Our results showed that the O_2_·^−^, H_2_O_2_, and MDA contents were sharply increased by Fe deficiency stress. This result agreed with that of Sun et al. [46], who reported that Fe deficiency could trigger ROS and H_2_O_2_ production at the early Fe-deficient stage in *M. xiaojinensis*. Our study also revealed that when exogenous Res was applied, the ROS level and MDA content decreased significantly. The activities of the antioxidant enzymes, such as SOD, POD, and CAT, increased significantly under Fe deficiency stress (Fig. S2). Exogenous Res can decrease the ROS level and MDA content in citrus seedlings by enhancing the activities of SOD, POD, and APX [27]. Our results revealed that Res had the same function as antioxidants under Fe deficiency stress.

Plant hormones are also involved in the Fe deficiency response. Exogenous IAA can regulate Fe uptake through the accumulation of NO under Fe deficiency stress [12]. OsARF16, a transcription factor that regulates auxin redistribution, is required for Fe deficiency response in rice (*Oryza sativa* L.) [47]. GA positively responds to Fe deficiency by regulating the expression of Fe-related genes, such as *bHLH038*, *bHLH039*, *FRO2*, and *IRT1* [14, 48]. Moreover, ABA and SA play a positive role in Fe deficiency response [15, 16]. In our study, the ABA and GA3 contents were significantly increased by Fe deficiency stress, whereas the IAA content was reduced. However, the IAA, ABA, and GA3 contents increased after the application of exogenous Res (Fig. 6). Meanwhile, our results also indicated that the application of exogenous Res decreased the content of DHZR and BL but produced no significant change in JA-Me (Fig. 6). CTK negatively regulates the root Fe uptake and inhibits the expression of *FRO2* and *IRT1* through a growth-dependent pathway in *Arabidopsis* [49]. JA and BR are also negatively regulated Fe homeostasis [17]. Exogenous BL decreases the expression of *OsNAS1*, *OsNAS2*, and *OsYSL2* in the stem and inhibit Fe transport in the phloem [13]. Overall, we inferred that aside from the Mechanism I strategy, exogenous Res could also enhance Fe uptake under Fe deficiency stress by increasing the contents of IAA, ABA, and GA3 and decreasing the contents of DHZR and BL.

In conclusion, our study found that exogenous Res significantly alleviated the Fe deficiency stress of the apple seedlings. The molecular and physiological mechanisms of such alleviation were examined. First, exogenous Res mainly improved the absorption of Fe by promoting the Mechanism I strategy. Second, Res functioned as an antioxidant to cope with the oxidative damage caused by Fe deficiency stress. Lastly, exogenous Res responded to Fe deficiency stress by regulating the contents of plant hormones indirectly. These findings provide a theoretical basis for analyzing how Res application improves the Fe deficiency tolerance of apples and elucidate the physiological role of Res under Fe deficiency stress.

## Supplementary Information

**Additional file 1:Figure S1** Effects of exogenous Res on apple seedlings under Fe deficiency stress in nutrient solution. (a) The phenotype resulting from the application of 100 µM exogenous Res to apple seedlings under Fe deficiency stress (The iron concentration was 4 µM, pH=5.9) at day 10. The apple seedlings in control group were cultured with complete nutrient solution (The iron concentration was 40 µM, pH=5.9). The etiolation rate (b) and fresh weight (c) of the apple seedlings after Fe deficiency and exogenous Res treatment for 10 days. Data represent the means ± SD of triplicate experiments. Different lowercase letters indicate significant differences, according to Fisher’s LSD (*P* < 0.05). **Figure S2** Effects of exogenous Res on oxidative damage and antioxidant enzyme activities under Fe deficiency stress. Effects of exogenous Res application on the levels of O_2_·^−^ and H_2_O_2_ (a) and MDA content (b) under Fe deficiency stress. Effects of exogenous Res application on the activities of SOD (c), POD (d) and CAT (e) under Fe deficiency stress. Data represent the means ± SD of triplicate experiments. Different lowercase letters indicate significant differences, according to Fisher’s LSD (*P* < 0.05). **Table S1 **The primers used for qRT-PCR.

## Data Availability

The datasets used and/or analysed during the current study are available from the corresponding author on reasonable request.

## Abbreviations

Res: Resveratrol
ROS: Reactive oxygen species
MDA: Malondialdehyde
SOD: Superoxide dismutase
POD: Peroxidase
CAT: Catalase
ABA: Abscisic acid
GA: Gibberellin
SA: Salicylic acid
JA: Jasmonate
CTK: Cytokinin
BL: Brassinolide
