# Supplementary material for: Resveratrol improves the iron deficiency adaptation of Malus baccata seedlings by regulating iron absorption
Source: BMC Plant Biol. 2021 Sep 23;21:433. doi: 10.1186/s12870-021-03215-y (PMC8459475; doi:10.1186/s12870-021-03215-y)
Supplement: Supplementary file 1 — Additional file 1:Figure S1 Effects of exogenous Res on apple seedlings under Fe deficiency stress in nutrient solution. (a) The phenotype resulting from the application of 100 µM exogenous Res to apple seedlings under Fe deficiency stress (The iron concentration was 4 µM, pH=5.9) at day 10. The apple seedlings in control group were cultured with complete nutrient solution (The iron concentration was 40 µM, pH=5.9). The etiolation rate (b) and fresh weight (c) of the apple seedlings after Fe deficiency and exogenous Res treatment for 10 days. Data represent the means ± SD of triplicate experiments. Different lowercase letters indicate significant differences, according to Fisher’s LSD (P < 0.05). Figure S2 Effects of exogenous Res on oxidative damage and antioxidant enzyme activities under Fe deficiency stress. Effects of exogenous Res application on the levels of O2·− and H2O2 (a) and MDA content (b) under Fe deficiency stress. Effects of exogenous Res application on the activities of SOD (c), POD (d) and CAT (e) under Fe deficiency stress. Data represent the means ± SD of triplicate experiments. Different lowercase letters indicate significant differences, according to Fisher’s LSD (P < 0.05). Table S1 The primers used for qRT-PCR. [file 12870_2021_3215_MOESM1_ESM.docx]

**Additional files**

**Additional file 1**

**
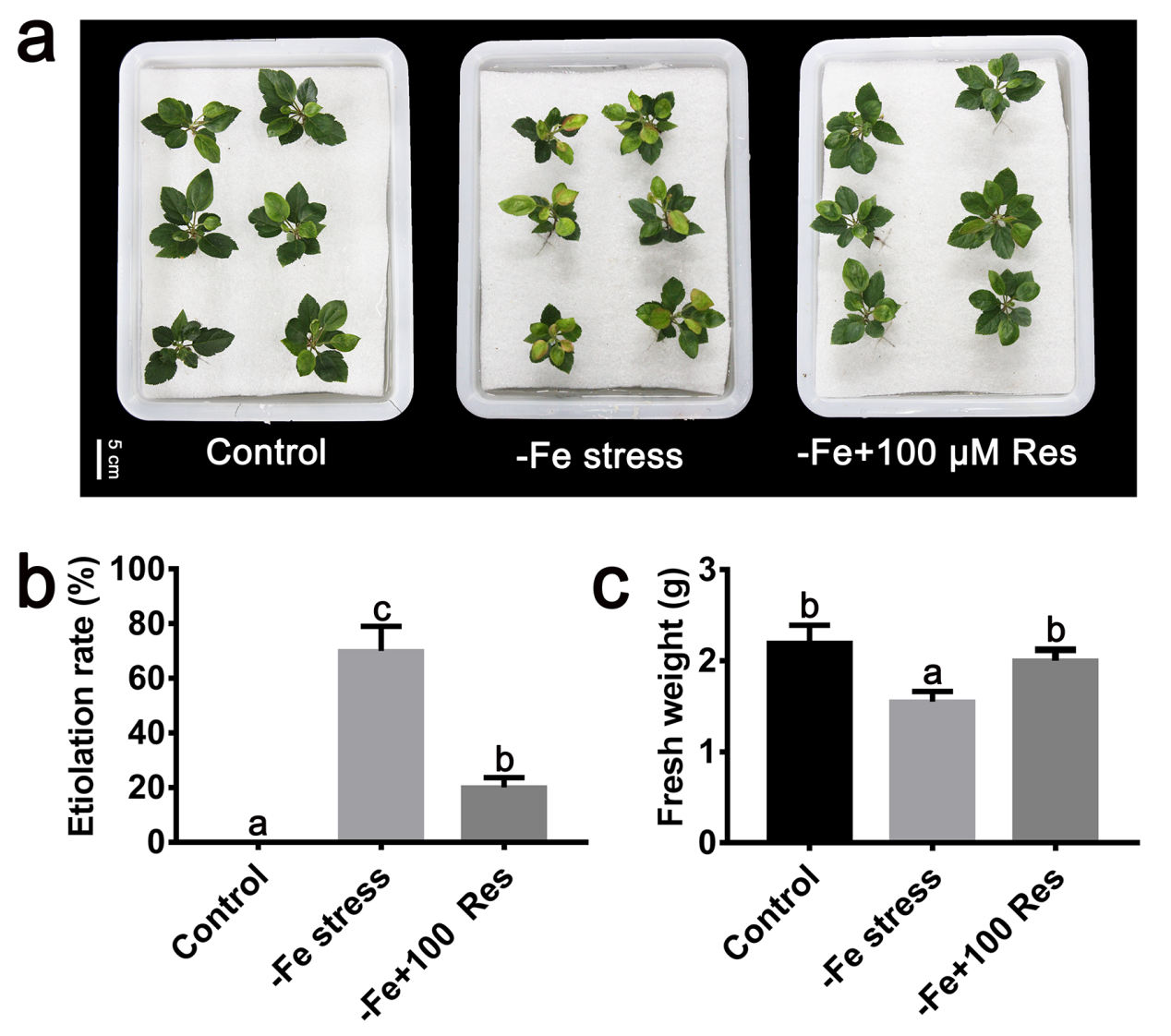
**

**Figure S1** Effects of exogenous Res on apple seedlings under Fe deficiency stress in nutrient solution. (a) The phenotype resulting from the application of 100 µM exogenous Res to apple seedlings under Fe deficiency stress (The iron concentration was 4 µM, pH=5.9) at day 10. The apple seedlings in control group were cultured with complete nutrient solution (The iron concentration was 40 µM, pH=5.9). The etiolation rate (b) and fresh weight (c) of the apple seedlings after Fe deficiency and exogenous Res treatment for 10 days. Data represent the means ± SD of triplicate experiments. Different lowercase letters indicate significant differences, according to Fisher’s LSD (*P* < 0.05).

**
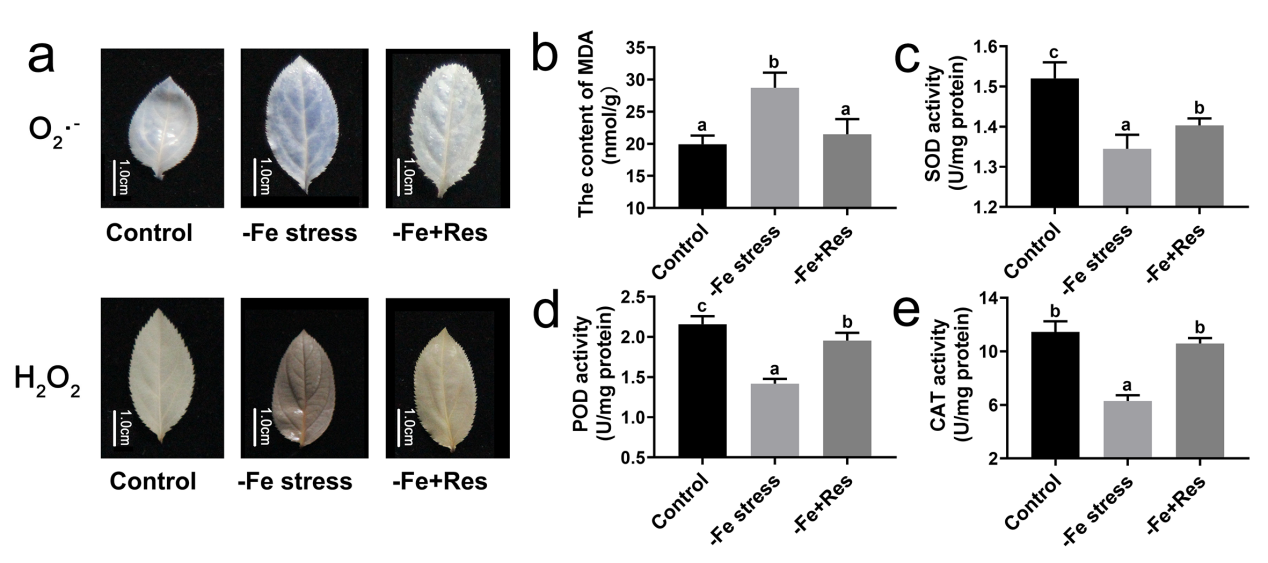
**

**Figure S2** Effects of exogenous Res on oxidative damage and antioxidant enzyme activities under Fe deficiency stress. Effects of exogenous Res application on the levels of O_2_·^−^ and H_2_O_2_ (a) and MDA content (b) under Fe deficiency stress. Effects of exogenous Res application on the activities of SOD (c), POD (d) and CAT (e) under Fe deficiency stress. Data represent the means ± SD of triplicate experiments. Different lowercase letters indicate significant differences, according to Fisher’s LSD (*P* < 0.05).

**Additional file 2**

**Table S1** The primers used for qRT-PCR

| Primers name | Primers sequence (5’→3’) |
| --- | --- |
| qAHA1-F | CCAGAGAAAACAAAAGAGAGTC |
| qAHA1-R | TTCACATTCACACCGAGATTG |
| qAHA3-F | TATTCTCTGCCTGTTGGTCATC |
| qAHA3-R | GCTCACTCCATTTTCCATCTCT |
| qAHA9-F | TTCATGGTCTTCAGCCACCT |
| qAHA9-R | TCACAACTGACTCGACGTGA |
| qFRO2-F | ACCGGACAGTGTCAATTTTAA |
| qFRO2-R | TGCTTTGTAAGTTGGGCATCC |
| qIRT1-F | TTTCTGGCTTTGTGGCTATGTT |
| qIRT1-R | GGCTGGAGTTTCACCATTATCT |
| qPYE1-F | TGAAGCTAGAGCGAATCAGTCG |
| qPYE1-R | CAAGGGTCCTGAAGGGTAAAAG |
| qBHLH104-F | CCAACGACACCCCTTCTTT |
| qBHLH104-R | CACTCCTTTTCCTGTGACTGTG |
| qBHLH105-F | GAAGGGACAGGCTAAATGACAA |
| qBHLH105-R | CTCAAACAAGAGGGAGAGAAGAG |
| qSIZ1-F | ATTCTAGAATGCAAATGCCATCC |
| qSIZ1-R | AGAGATTCTATTCAGAGTCCGAG |
| Actin-F | CTTCAATGTGCCTGCCATGTAT |
| Actin-R | AATTTCCCGTTCAGCAGTAGTG |
